# Estimating genetic parameters of digital behavior traits and their relationship with production traits in purebred pigs

**DOI:** 10.1186/s12711-024-00902-w

**Published:** 2024-04-16

**Authors:** Mary Kate Hollifield, Ching-Yi Chen, Eric Psota, Justin Holl, Daniela Lourenco, Ignacy Misztal

**Affiliations:** 1 Department of Animal and Dairy Science, University of Georgia, Athens, GA USA; 2 Genus PIC, Hendersonville, TN USA

## Abstract

**Background:**

With the introduction of digital phenotyping and high-throughput data, traits that were previously difficult or impossible to measure directly have become easily accessible, offering the opportunity to enhance the efficiency and rate of genetic gain in animal production. It is of interest to assess how behavioral traits are indirectly related to the production traits during the performance testing period. The aim of this study was to assess the quality of behavior data extracted from day-wise video recordings and estimate the genetic parameters of behavior traits and their phenotypic and genetic correlations with production traits in pigs. Behavior was recorded for 70 days after on-test at about 10 weeks of age and ended at off-test for 2008 female purebred pigs, totaling 119,812 day-wise records. Behavior traits included time spent eating, drinking, laterally lying, sternally lying, sitting, standing, and meters of distance traveled. A quality control procedure was created for algorithm training and adjustment, standardizing recording hours, removing culled animals, and filtering unrealistic records.

**Results:**

Production traits included average daily gain (ADG), back fat thickness (BF), and loin depth (LD). Single-trait linear models were used to estimate heritabilities of the behavior traits and two-trait linear models were used to estimate genetic correlations between behavior and production traits. The results indicated that all behavior traits are heritable, with heritability estimates ranging from 0.19 to 0.57, and showed low-to-moderate phenotypic and genetic correlations with production traits. Two-trait linear models were also used to compare traits at different intervals of the recording period. To analyze the redundancies in behavior data during the recording period, the averages of various recording time intervals for the behavior and production traits were compared. Overall, the average of the 55- to 68-day recording interval had the strongest phenotypic and genetic correlation estimates with the production traits.

**Conclusions:**

Digital phenotyping is a new and low-cost method to record behavior phenotypes, but thorough data cleaning procedures are needed. Evaluating behavioral traits at different time intervals offers a deeper insight into their changes throughout the growth periods and their relationship with production traits, which may be recorded at a less frequent basis.

## Background

High-throughput phenotyping, digital data recording, and novel traits have recently become topics of interest in animal production. With advancements in technology, phenotypes can be collected with higher accuracy, in greater quantities, and new traits that are difficult or impossible to measure directly can be captured [1]. Applications include sensors, wearable technology, imaging, video, and audio recording to assess body temperature [2], stress [3], disease [4, 5], behavior [6], or overall health [7, 8].

In pork production, meat quality and quantity are economically relevant traits that are under genetic or genomic selection. Determining the meat characteristics of an animal may not be possible until the peak of production age or slaughter [9]. If a trait that can be measured early in an animal’s life is indicative of later production traits, it allows for earlier selection and culling decisions, which can reduce the generation interval. In addition, incorporating phenotypes from progeny at the multiplication or commercial level would benefit nucleus level parents and enhance accuracy of the genomic estimated breeding values (GEBV) of the elite animals. As collecting phenotypes can be costly and labor-intensive, automated data collection via digital phenotyping could increase data collection at a low cost and with more precision than human labor [10, 11]. Traits that are of interest to capture using digital phenotyping are those that are heritable and that are genetically related to, or that affect an animal’s economically relevant production traits.

Animal behavior is one example of such a trait but recording behavior using cameras is challenging due to the difficulty in identifying individual animals. Technologies to obtain automated long-term individualized behavior data include the use of radio frequency identification (RFID) [12], ultra-wideband [13], and visual fingerprinting [14] for animal recognition in a pen. However, these methods are fundamentally limited in spatial resolution (RFID and ultra-wideband) and reliability (visual fingerprinting). An alternative approach that provides reliable identification relies on industry-standard ear tags, albeit intermittently, i.e. when the ear tag is exposed to the camera [15]. Regardless of which method is used, thorough data cleaning is always necessary to ensure that the information captured is realistic and accurate.

Behavior traits that are genetically correlated to production traits can be used for genetic improvement, including activity levels [16], eating patterns [17], and management refinement [18]. The goal of this study was to create a data quality control procedure and investigate behavior traits that can be captured by digital phenotyping and their phenotypic and genetic correlations with production traits.

## Methods

### Dataset

The data were provided by PIC (Genus Company, Hendersonville, TN) and included 119,812 day-wise behavior records for 2008 pigs collected between August 26, 2021, and May 23, 2023. All animals were housed on the same farm and belonged to two lines of purebred pigs. Digital behavior phenotypes were extracted from video recordings and included the daily cumulative time each animal spent eating, drinking, lying laterally, lying sternally, sitting, and standing, and the distance traveled. Whereas, standing refers to the raised position which also includes walking or running. The recording period began after the on-test, at about 10 weeks of age, and ended at off-test, for 70 recording days. There were 12 cameras that recorded 14 h per day (5:00 a.m. to 7:00 p.m.), with one camera per pen and two pens per room had cameras. The two cameras in the same room recorded simultaneously throughout the recording period. The recording group was defined as the animals under the same camera with the same recording start date.

All video was processed by a multi-object tracking algorithm to extract individualized activity data. The first stage of processing consisted of detecting individual pigs using a customized version of the DeepCut pose estimation algorithm [19] that detects mid-points, snouts, and right ear tag locations and associated these with individuals. For each detected midpoint, a convolutional neural network (CNN) also estimated the posture of the pig and whether the pig was eating. Snout locations were used to limit the possible locations where eating can take place and to estimate drinking activities based on proximity to the feeder and waterer, respectively.

Once detected, each pig was tracked using Hungarian matching [20] to follow detected pigs from one frame to the next, with pigs that were not detected assumed to “stay put” in their previous locations. The most challenging aspect of reliable tracking is maintaining identity, for which a custom ear tag reading method developed by PIC was used [15]. This method allows tags to be read at low resolution with challenging perspectives, motion blur, noise, and shadows.

Of the 2008 animals with digital behavior records, 1705 had production trait records. The production traits included average daily gain (ADG), back fat thickness (BF), loin depth (LD). All production traits were captured at off-test, at about 20 weeks of age, i.e. at the end of the recording period.

To validate the system’s accuracy in determining location, posture, and identification, a trial with 36 randomly selected pigs was conducted (six from each of six pens, with each pen housing 19 pigs). These pigs were distinctly marked for easy identification in video footage. Across 5 days, 330 annotated images were produced by the tracking algorithm to highlight each pig's location, posture, and ear tag identification. The cross-checker was tasked with first identifying the pig with specific paint markings in the image. If the pig could not be reliably identified, this image was not included in the analysis. If it could be identified with high confidence, its identity was compared to the known identity from the table of paint-ID correspondences. Its posture and eating/drinking status were also manually recorded and compared to the automated activity detections. Overall, the pigs of interest were all identified in more than 95% of the images and used to evaluate accuracy. Preliminary validation metrics precision, recall, and F1-score indicated an accuracy of correctly annotating location, posture, and identity greater than 97% [21].

### Data cleaning procedure

Since digital phenotyping is a recently developed and evolving data collection technique, quality control efforts are needed. After analyzing the data patterns, several observations were made that suggested that, under certain conditions, the data may not be reliable. As the equipment was set-up and calibrated, and the farm standard operating procedures and data-extracting algorithms were being created and adjusted, a “learning period” was designated. The “learning period” spanned from the beginning of the recording period, August 21, 2021, to March 17, 2022, and the data collected during this period were discarded from the analyses. After the learning period, the average recording time per day by each camera became more consistent, and the start and end dates for each 70-day recording group became more cyclic than before the learning period.

The data for each pig and each day were summarized into cumulative time spent in each behavior, position, or distance traveled over a 14-h period. Therefore, days with less than 8 h of recording time were removed as this is not representative of the behavior for the total 14 h. Days with less than 14 h and more than 8 h of recording time were scaled up to 14 h by dividing the daily record by the number of recording hours and multiplying by 14. The data for culled animals on the day they were extracted from the pen were removed, as the time when an animal was extracted from the pen was not available, so it is not known how many hours to account for the culled animal’s activity for that day. The start and end days of the recording period were also removed from the analyses due to the lack of the full 14 recording hours and disruptions from loading and unloading the animals.

After further examination of the data, some records for distance traveled were biologically impossible compared to the time spent standing. For example, one animal stood for only three min and was recorded to have traveled 600 m. After investigation, it was determined that the data-extracting algorithm accumulated meters traveled if the animal was rotating while in a sitting position. To account for this, the daily distance data were truncated to 15 m per min standing, and all daily records that exceeded this ratio were discarded. The data extracting algorithm will be modified for future studies to prevent recording distance while the animal is in the sitting position.

**Fig. 1:**
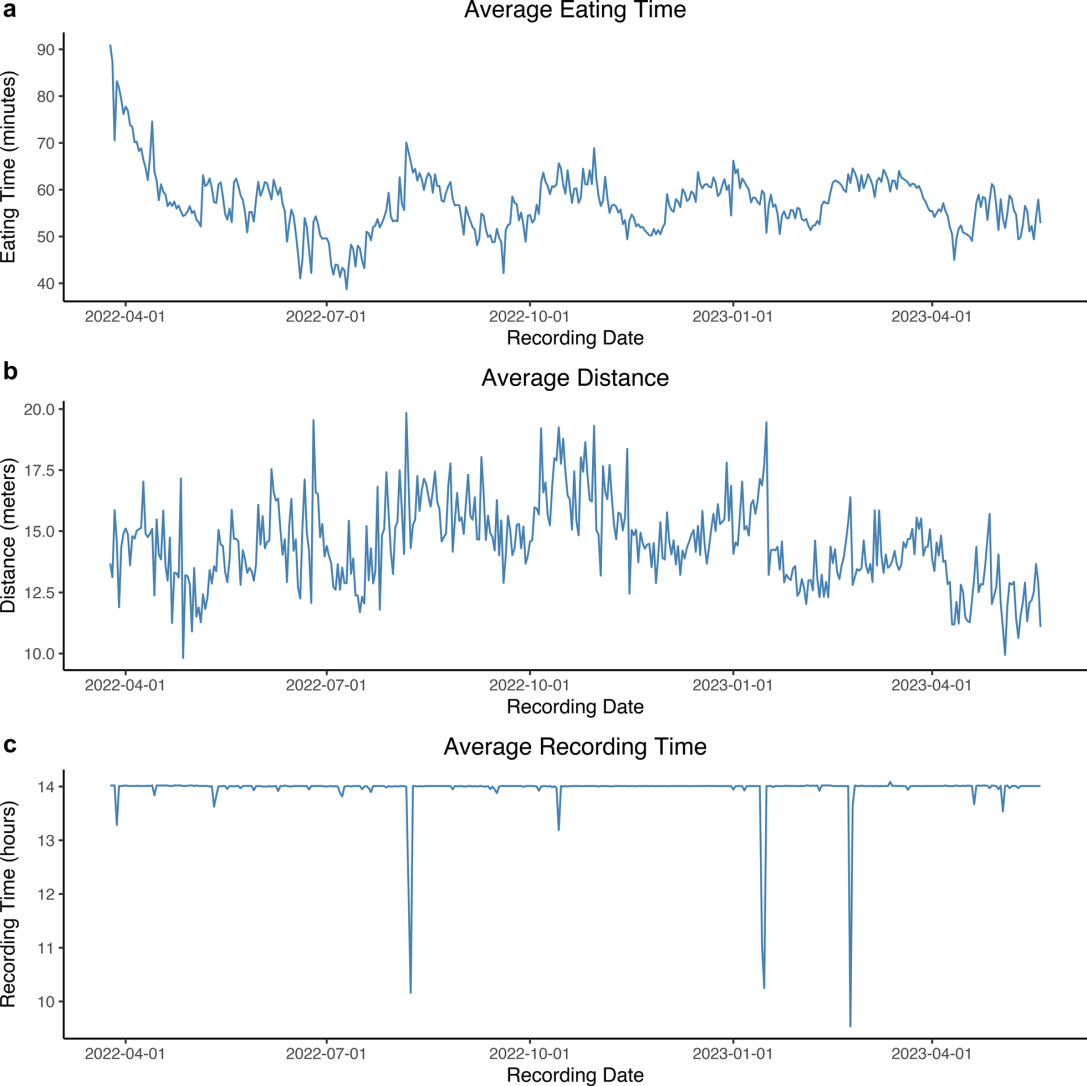
Average eating, distance, and recording time per group over time. **a** Average eating time in min, **b** Average distance in m and **c** Average recording time in h

After data cleaning, 77,423 daily records from 1327 animals remained. The average eating time, distance, and recording time per day after the learning period and following data cleaning are shown in Fig. 1a–c, and the summary statistics for the behavior traits are in Table 1. Since the data in Fig. 1a–c are averaged over the recording days, they include animals of various ages and at different stages of the recording period. The peak shown in the average eating time is because, at that time, only young pigs were recorded and they spend more time at the feeder than older pigs. It should be noted that the drinking time behavior measures time spent at the waterer, not the amount of water consumed.

**Table 1:** Summary statistics for digital behavior traits after data cleaning

Trait	Mean	Median	SD	Min	Max
Eating time	56.41	54.41	19.55	0.00	165.96
Drinking time	7.25	6.36	4.51	0.0	84.65
Laterally lying time	287.52	285.45	90.81	6.06	718.70
Sternally lying time	359.12	358.20	73.59	79.84	716.75
Sitting time	22.97	18.06	18.13	0.00	260.19
Standing time	170.28	168.60	52.21	0.00	611.34
Distance (m)	872.55	827.31	357.47	0.00	3589.54

Traits recorded in time are shown in min

*SD* standard deviation

Only animals with off-test production records were used to estimate genetic parameters, which included 71,999 daily behavior records from 1079 animals, among which 563 were of one line and 516 were of a different line. Summary statistics for the production traits of these animals are in Table 2.

**Table 2:** Summary statistics for production traits

Trait	Mean	Median	SD	Min	Max
ADG, g	710.59	710.77	63.98	510.90	934.00
BF, mm	8.33	8.00	2.30	4.58	17.12
LD, mm	66.34	66.10	5.40	51.50	83.10

*ADG* average daily gain, *BF* back fat thickness, *LD* loin depth, *SD* standard deviation

### Models and statistical analyses

Variance components were estimated using the blupf90+ program [22, 23] that applies a single-trait and a two-trait linear model. The equation for all models can be expressed as:

$${\mathbf{y}} = {\mathbf{X}} {\varvec{\upbeta}} + {\mathbf{Zu}} + {\mathbf{W}}_{\varvec{1}} {\mathbf{l}} + {\mathbf{W}}_{\varvec{2}} {\mathbf{c}} + {\mathbf{e}},$$

where $\mathbf{y}$ is the vector of phenotypes, ${\varvec{\upbeta}}$ is the vector of the fixed line effects, $\mathbf{u}$, $\mathbf{l}$, and $\mathbf{c}$ are random vectors of additive genetic, common litter, and contemporary group effects, respectively. Contemporary groups were represented by off-test day and year. A pen or camera effect was not included in the model because it was confounded with the litter effect. Elements of $\mathbf{y}$ are related to elements $\mathbf{u}$, $\mathbf{l}$, and $\mathbf{c}$ by incidence matrices $\mathbf{Z}$, ${\mathbf{W}}_{\varvec{1}}$, and ${\mathbf{W}}_{\varvec{2}}$, respectively, and $\mathbf{e}$ is a random vector of residuals. For single-trait models, the covariance matrices were assumed to be:

$${\text{Var}}\left[ {\begin{array}{*{20}c} {\mathbf{u}} \\ {\mathbf{l}} \\ {\mathbf{c}} \\ {\mathbf{e}} \\ \end{array} } \right] = \left[ {\begin{array}{*{20}c} {{\mathbf{A}}{\upsigma }_{{\text{u}}}^{2} } & 0 & 0 & 0 \\ 0 & {{\mathbf{I}}{\upsigma }_{{\text{l}}}^{2} } & 0 & 0 \\ 0 & 0 & {{\mathbf{I}}{\upsigma }_{{\text{c}}}^{2} } & 0 \\ 0 & 0 & 0 & {{\mathbf{I}}{\upsigma }_{{\text{e}}}^{2} } \\ \end{array} } \right],$$

where $\mathbf{A}$ is the numerator relationship matrix, $\mathbf{I}$ is the identity matrix, and ${\upsigma }_{{\text{u}}}^{2}$, ${\upsigma }_{{\text{l}}}^{2}$, ${\upsigma }_{{\text{c}}}^{2}$, and ${\upsigma }_{{\text{e}}}^{2}$ are variances for the additive genetic, common litter, contemporary group, and residual effects, respectively.

For two-trait models, the vectors $\mathbf{u}$, $\mathbf{l}$, and $\mathbf{c}$, and $\mathbf{e}$ were assumed to be distributed as multivariate normal with mean zero and the following covariance structure:

$${\text{Var}}\left[ {\begin{array}{*{20}c} {\mathbf{u}} \\ {\mathbf{l}} \\ {\mathbf{c}} \\ {\mathbf{e}} \\ \end{array} } \right] = \left[ {\begin{array}{*{20}c} {{\mathbf{A}} \otimes {\mathbf{G}}} & 0 & 0 & 0 \\ 0 & {{\mathbf{I}} \otimes {\mathbf{L}}} & 0 & 0 \\ 0 & 0 & {{\mathbf{I}} \otimes {\mathbf{C}}} & 0 \\ 0 & 0 & 0 & {{\mathbf{I}} \otimes {\mathbf{R}}} \\ \end{array} } \right],$$

where $\mathbf{G}$ is the additive genetic (co)variance matrix between the two traits, $\mathbf{L}$ is the common litter (co)variance matrix, $\mathbf{C}$ is the contemporary group (co)variance matrix, and $\mathbf{R}$ is the residual (co)variance matrix. Single-trait models were used for heritability estimation for the behavior traits. Two-trait models were used to analyze the relationship between behavior and production traits and to determine the redundancy in the 70 recording days by splitting the recording time into separate periods and determining the relationship of behavior traits in each period with those for the full recording time or with a production trait.

## Results and discussion

### Behavior trends

**Fig. 2:**
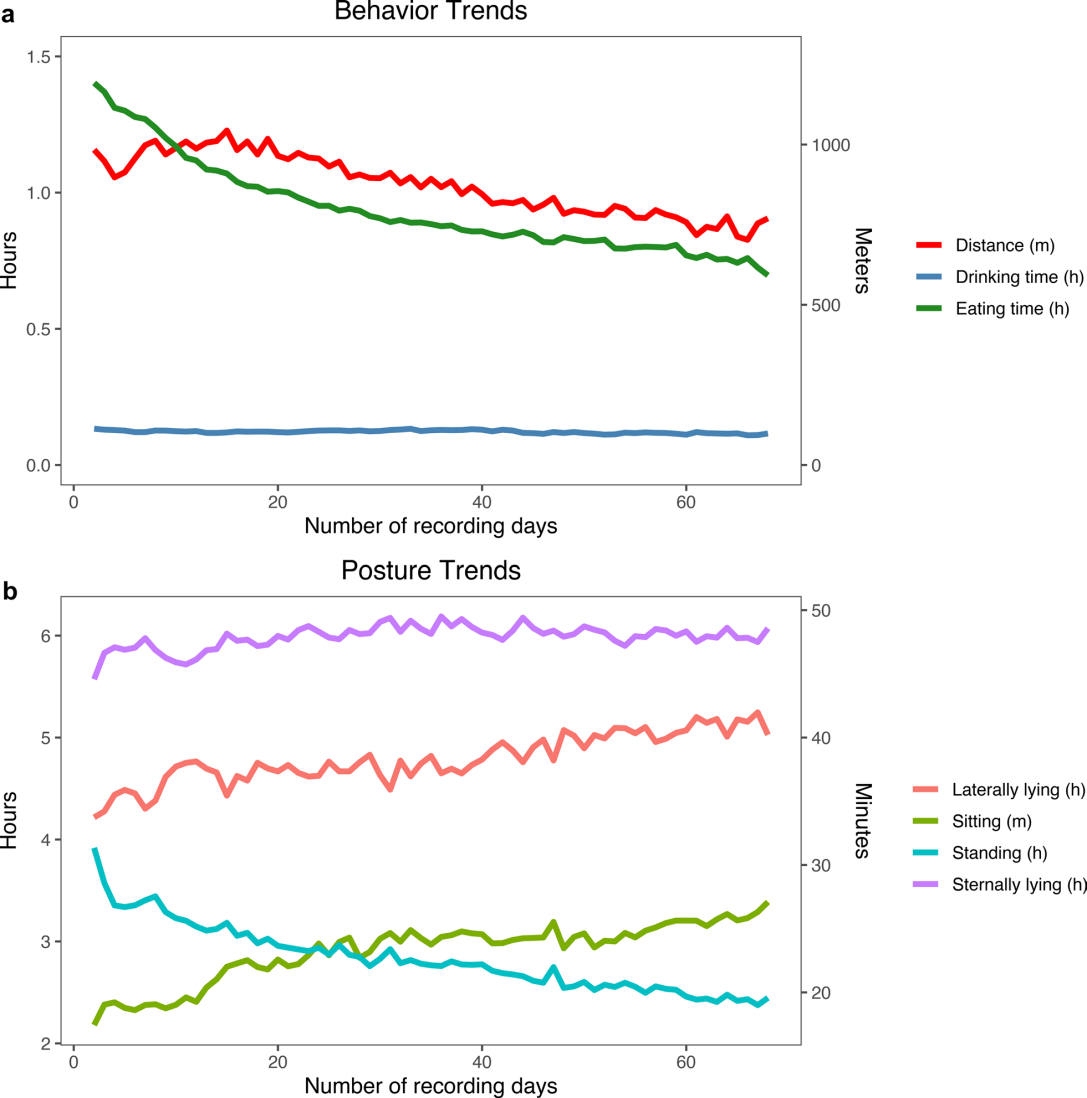
Average behavior and posture trends per individual over time. Eating time, drinking time, laterally lying, standing, and sternally lying are shown in h. Sitting is shown in min and distance is shown in m. **a** Behavior trends and **b** Posture trends

**Fig. 3:**
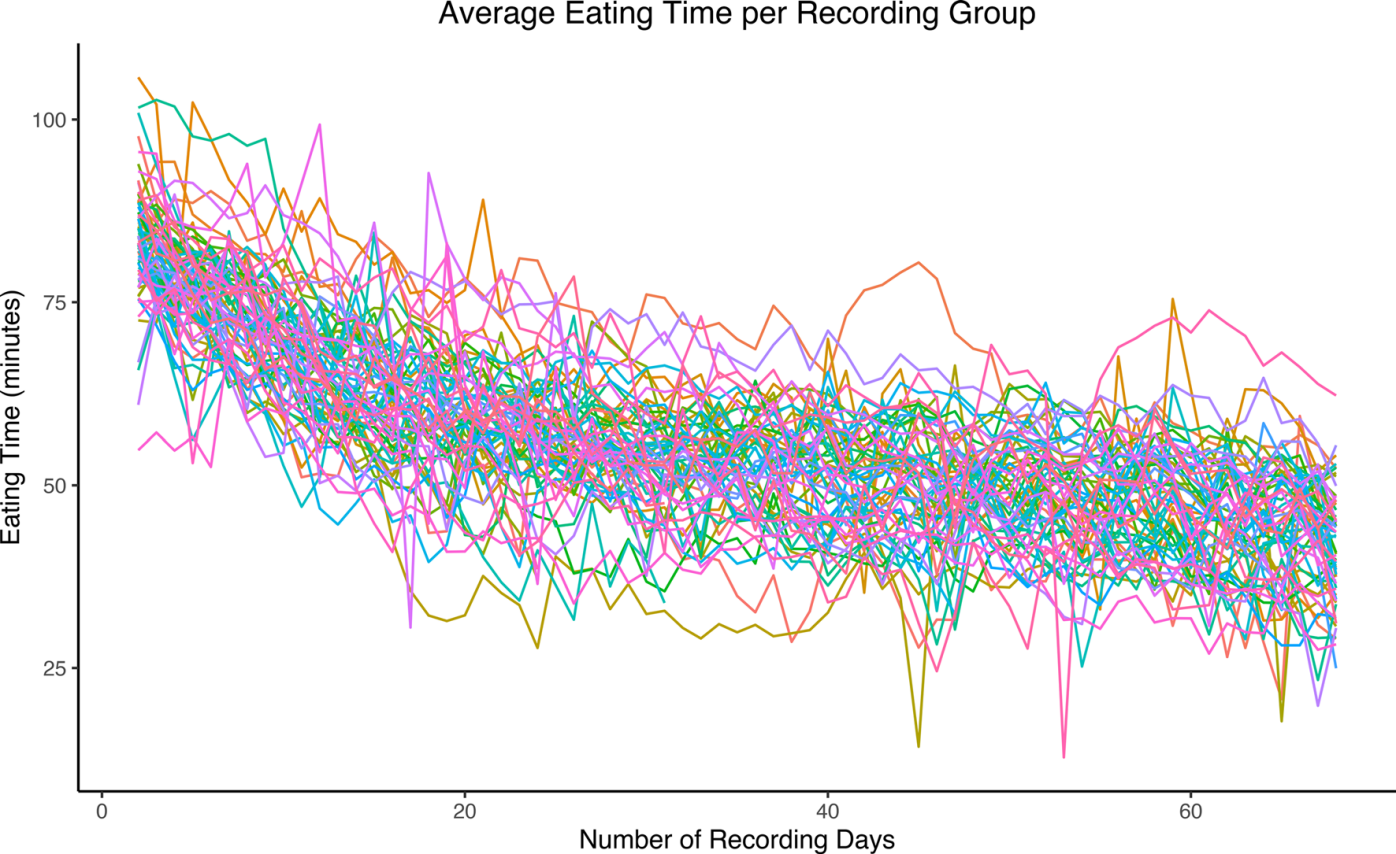
Average eating time per group over time

**Fig. 4:**
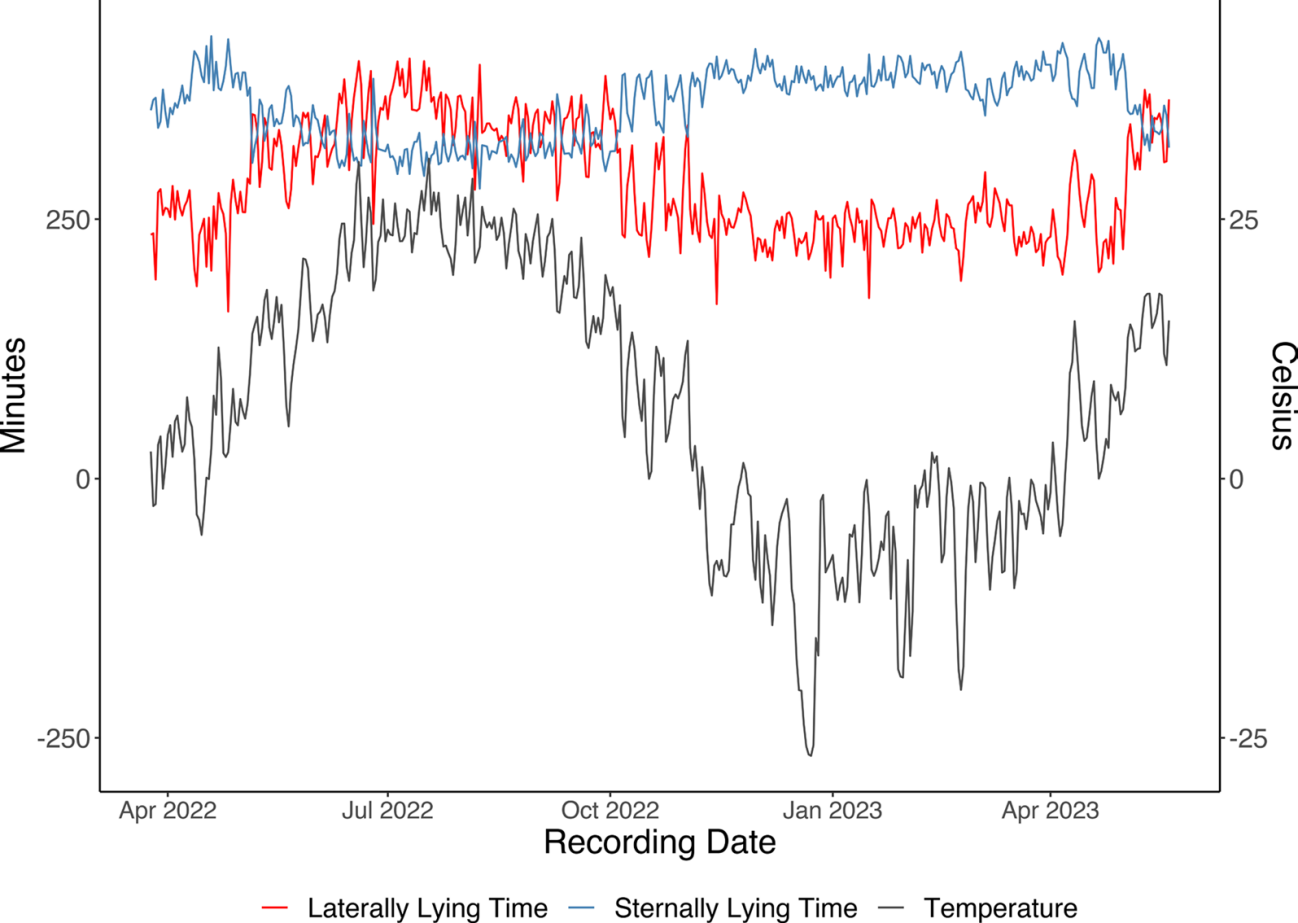
Average lateral lying time, sternal lying time, and temperature over time. The temperature data were obtained from the NASA POWER website (https://power.larc.nasa.gov/data-access-viewer/) using the longitude and latitude coordinates of the farm and were the average air temperatures at a height of two m above the surface of the earth

All results shown are with data after the learning period and cleaning. Trends in average behavior and posture over the recording period based on the clean data for animals with off-test records are shown in Fig. 2a and b. The data showed a decreasing pattern for eating time, distance traveled, and standing time as the pigs aged. These trends agree with Hyun and Ellis [24], who showed that younger pigs eat more meals per day and eat less per meal than older pigs. A slight increasing pattern was seen for time lying laterally and sternally as pigs aged. Figure 3 shows the average eating time per recording group over the recording period, where each line is a unique recording group. The substantial variation in behavior between recording groups is shown as in eating time trends in Fig. 3. Therefore, it is important to have an adequate data cleaning procedure and statistical model to separate genuine variation between groups from noise. We also observed that the pigs spent more time lying laterally and less time lying sternally in the warmer months than in the cooler months, which agrees with studies from Ekkel et al. [25] and Huynh et al. [26]. The trends of the average lateral lying time, sternal lying time, and temperature over the recording date are shown in Fig. 4. The temperature data were retrieved from the NASA POWER website (https://power.larc.nasa.gov/data-access-viewer/) using the longitude and latitude coordinates of the farm and reflect the average outside air temperature at a height of two meters. Similarly, Aarnink et al. [27] found that the relative number of pigs that lay laterally increased by 1.8% for each degree Celsius rise in temperature. There was no pattern seen for drinking time as the pigs aged.

### Behavior trait heritabilities and correlations

Table 3 shows estimates of phenotypic and genetic correlations, and of heritabilities. Eating time had the highest heritability estimate among the behavior traits, at 0.57, with a standard error of 0.12. The behavior trait with the lowest heritability estimate was laterally lying at 0.19, with a standard error of 0.07. The standard errors of the heritability estimates of the behavior traits were sizeable, ranging from 0.07 to 0.13, likely observed due to the small size of the dataset and the lack of multiple generations with records. The behavior traits with the strongest phenotypic correlation estimates were standing time and distance (0.67) and laterally lying time and sternally lying time (− 0.82). The same trait combinations had the strongest genetic correlation estimates, i.e. 0.93 ± 0.03 for standing time and distance and − 0.84 ± 0.04 for laterally lying time and sternally lying time. These estimates are expected, as generally, the animals are in a standing position while mobile and may prefer one lying position over the other, especially if the 70 days of recording time is during consistently hot or cold weather.

**Table 3:** Estimates of heritabilities and standard errors using single-trait models (diagonal) and of phenotypic (upper diagonal) and genetic correlations with standard errors (lower diagonal) using two trait models

	Eating time	Drinking time	Laterally lying time	Sternally lying time	Sitting time	Standing time	Distance (m)	ADG	BF	LD
Eating time	0.57 ± 0.12	0.17	− 0.34	0.04	0.08	0.51	0.18	0.11	0.08	0.09
Drinking time	0.38 ± 0.12	0.38 ± 0.12	0.15	− 0.33	0.01	0.23	0.28	− 0.03	0.08	− 0.13
Laterally lying time	− 0.40 ± 0.16	− 0.33 ± 0.14	0.19 ± 0.07	− 0.82	− 0.26	− 0.47	− 0.06	− 0.11	0.00	− 0.21
Sternally lying time	− 0.41 ± 0.08	− 0.43 ± 0.31	− 0.84 ± 0.04	0.22 ± 0.08	0.12	− 0.07	− 0.36	0.20	0.01	0.29
Sitting time	0.01 ± 0.07	0.26 ± 0.09	− 0.23 ± 0.08	− 0.25 ± 0.19	0.48 ± 0.13	− 0.13	− 0.09	0.15	− 0.03	0.11
Standing time	0.69 ± 0.06	0.62 ± 0.11	− 0.72 ± 0.10	− 0.62 ± 0.08	− 0.48 ± 0.08	0.43 ± 0.11	0.67	− 0.16	0.00	− 0.09
Distance (m)	0.45 ± 0.09	0.44 ± 0.13	− 0.68 ± 0.11	− 0.58 ± 0.09	− 0.05 ± 1.00	0.93 ± 0.03	0.38 ± 0.10	− 0.30	− 0.01	− 0.23
ADG	0.14 ± 0.15	0.32 ± 0.22	0.50 ± 0.18	− 0.10 ± 0.16	0.26 ± 0.16	− 0.56 ± 0.11	− 0.57 ± 0.10	0.38 ± 0.12	0.29	0.52
BF	0.18 ± 0.07	0.18 ± 0.02	0.19 ± 0.08	− 0.04 ± 0.09	0.00 ± 0.08	− 0.17 ± 0.07	− 0.27 ± 0.09	0.56 ± 0.08	0.53 ± 0.11	0.10
LD	0.04 ± 0.09	− 0.07 ± 0.11	0.24 ± 0.12	0.09 ± 0.12	0.03 ± 0.03	− 0.37 ± 0.10	− 0.48 ± 0.14	0.84 ± 0.08	0.26 ± 0.20	0.30 ± 0.10

The behavior traits were averaged over the total recording period. All traits based on time were expressed in min

*ADG* average daily gain, *BF* back fat thickness, *LD* loin depth

### Redundancy in recording time

**Fig. 5:**
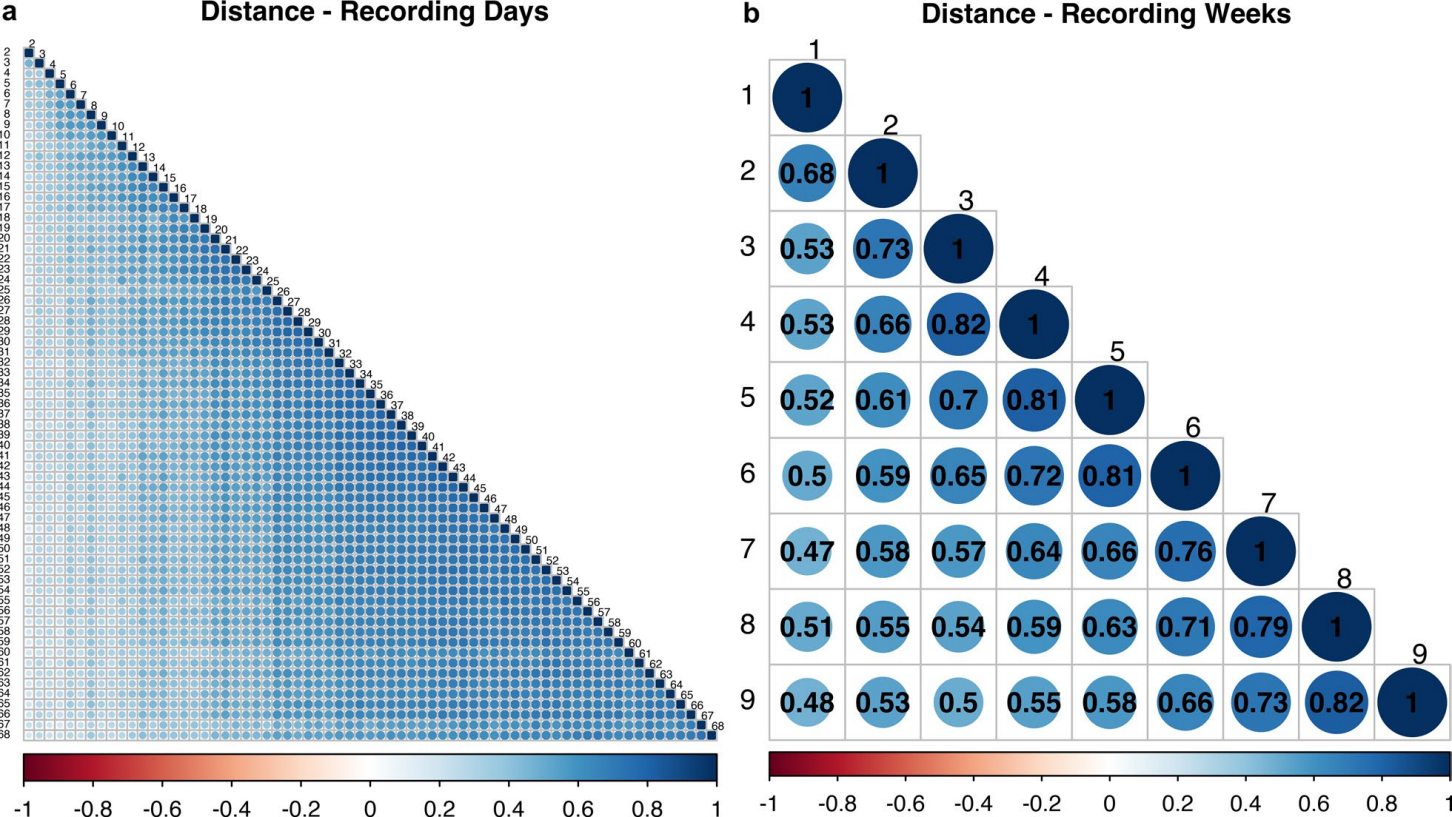
Estimates of phenotypic correlations of average daily and weekly distance traveled. The data used are from the dataset after the data cleaning procedure and for animals with off-test records. **a** Average daily distance traveled and **b** Average weekly distance traveled

To determine whether all 70 days of recording time were necessary, we analyzed phenotypic correlations of daily and weekly intervals for the same trait and fitted two-trait models with a time interval of a behavior trait as one trait and either the total average of the behavior trait or a production trait as the second trait. In general, estimates of phenotypic correlations between daily and weekly intervals of the same trait became stronger as the recording time progressed and were stronger closer to the end of the recording period compared to the beginning. This suggests that the animals behaved more similarly as they aged and adapted to their environment, which infers redundant information. For example, Fig. 5a and b show estimates of phenotypic correlations between daily and weekly averages for distance traveled; the correlation for weeks 1 and 2 was 0.68, while the correlation for weeks 8 and 9 was 0.82. Days closer to the end of the recording period had stronger correlations than days at the beginning of the recording period. Thus, not all 70 recording days are needed to capture the behavior of the animals or to associate the production traits with behaviors.

**Fig. 6:**
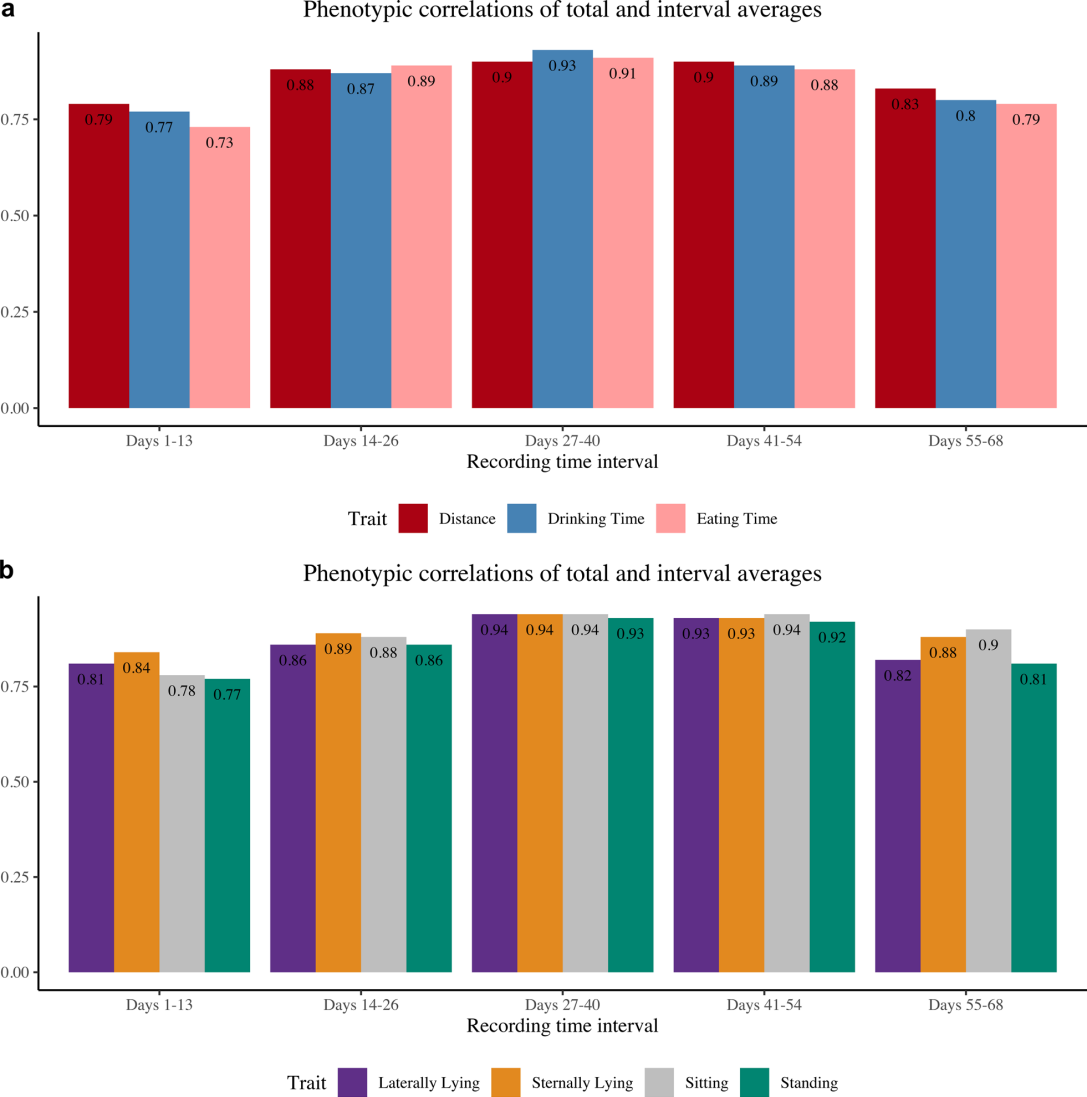
Estimates of phenotypic correlations between the total average and the average of each recording time interval for each behavior trait. All traits are expressed in min, expect for distance which is expressed in m. **a** Traits: distance traveled, drinking time, eating time and **b** Traits: laterally lying time, sternally lying time, sitting time, standing time

**Fig. 7:**
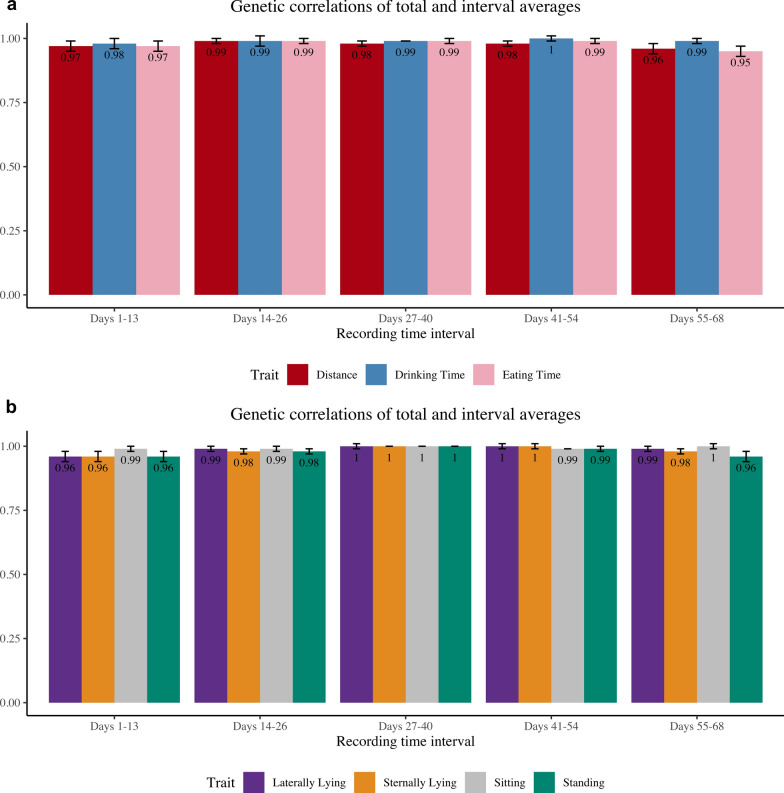
Estimates of genetic correlations (standard errors) between the total average and the average of each recording time interval for each behavior trait. All traits are expressed in min, expect for distance which is expressed in m. **a** Traits: distance traveled, drinking time, eating time and **b** traits: laterally lying time, sternally lying time, sitting time, standing time

For the two-trait models, the behavior traits were split into five intervals: days 1–13, 14–26, 27–40, 41–54, and 55–68. Estimates of phenotypic and genetic correlations were compared between the two traits and are shown in Figs. 6a and b and 7a and b, respectively. For all traits, generally, the middle intervals had higher correlations with the total average compared to intervals at the beginning and end of the recording time, and estimates of genetic correlations were higher than estimates of phenotypic correlations. For phenotypic and genetic correlations between intervals and total recording period average, eating time had the lowest phenotypic correlation estimate, at 0.73 for the 1- to 13-day interval, and the lowest genetic correlation estimate at 0.95 for the 55-to 68-day interval, respectively. The highest phenotypic correlations were 0.94, for laterally lying and sternally lying, and sitting for the 27- to 40-day interval and 0.94 for sitting for the 41-to 54-day interval. The highest genetic correlation estimates were 1.00 for laterally lying, sternally lying, sitting, and standing for the 27- to 40-day interval, 1.00 for laterally lying, sternally lying, and drinking time for the 41- to 54-day interval, and 1.00 for sitting for the 55- to 68-day interval.

A genetic correlation of 1.00 indicates that the two traits have the same genetic basis. Therefore, if recording is to capture the average behavior of the animals from the on-test period until the off-test period, then the same information, or the most informative data, can be captured during days 27–40 of this period, as this interval has a 1.00 genetic correlation with the total average of the recording period. As storing videos is very costly, recording for the entire 70 days is not necessary, as the behavior of the animal becomes redundant, and the overall behavior patterns can be captured in a span of 2 weeks. The closer the genetic correlations are to 0, the weaker the relationship between the two traits, indicating that the traits give different genetic information.

### Relationships between behavior and production traits

The purpose of this study was to determine if a relationship exists between an animal’s behavior and its production performance. If a sufficiently strong relationship exists, the behavior data could predict the production trait phenotypes before the animal’s off-test. Two-trait models were fit to estimate the relationship between behavior traits and production traits. To determine if the behavior during a specific time span in the recording period had a stronger relationship with the production traits than the average behavior trait for the entire period, behavior traits measured over five recording intervals were analyzed also in two-trait models with production traits.

**Fig. 8:**
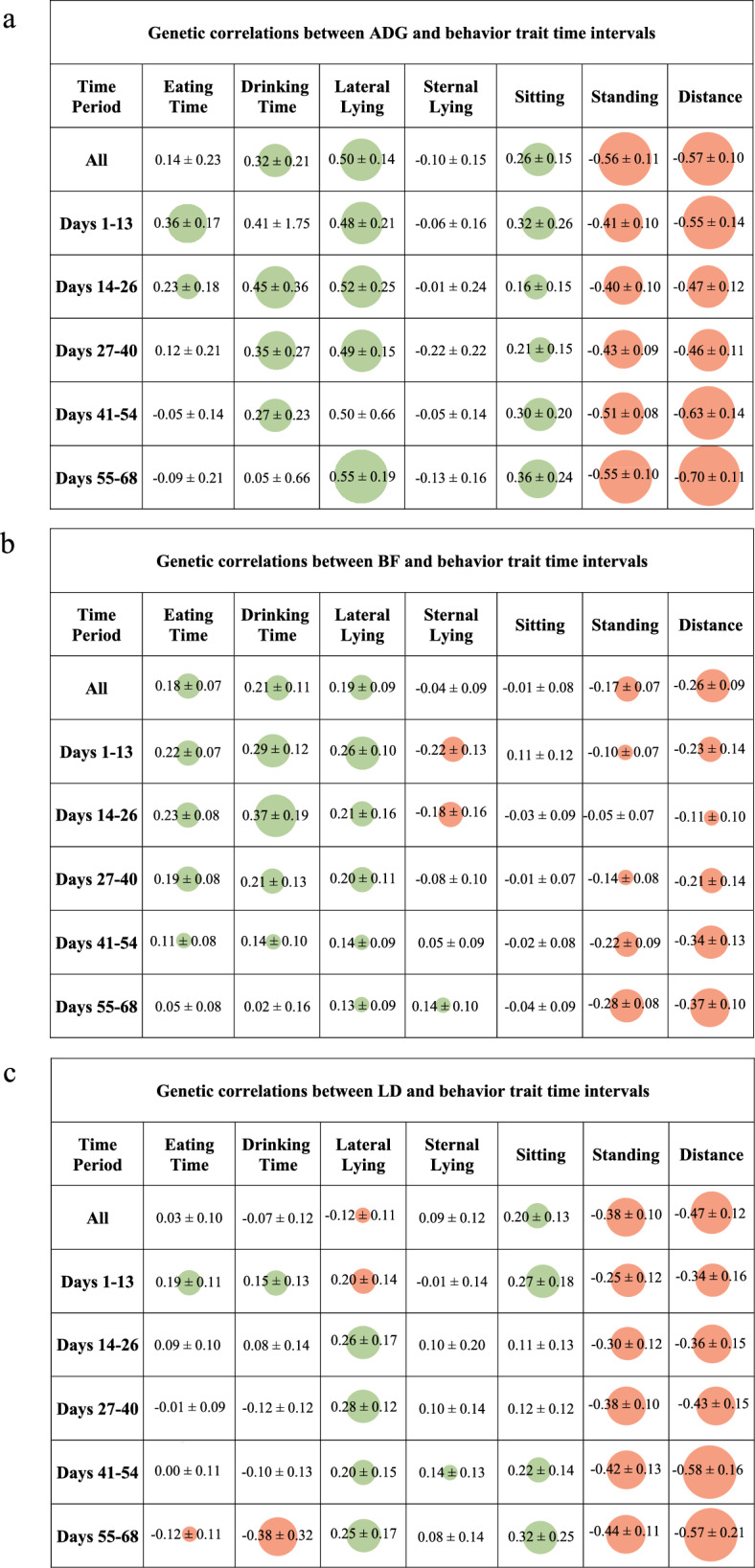
Estimates of genetic correlations (standard errors) between production traits, and time intervals or total averages of each behavior trait. The cells with green circles denote a positive correlation, whereas the cells with red circles denote a negative correlation and the size of the circle indicates the strength of the correlation. The cells without color indicate a range of genetic correlations that passed through 0.0 when the standard error was considered. **a** ADG, **b** BF and **c** LD

The strongest positive genetic and phenotypic correlations between average behavior across the full recording period and production traits were estimated for lateral lying time and ADG (0.50 ± 0.18) and sternal lying time and LD (0.29), respectively (Fig. 8a–c). The strongest negative genetic and phenotypic correlations were estimated between distance and ADG (− 0.57 ± 0.10 and -0.30, respectively). The strongest positive genetic correlation between average behavior traits for the five time periods and production traits was estimated between the average lateral lying time for the 55–68-day interval and ADG (0.55 ± 0.19). The strongest negative genetic correlation was estimated between average distance for the 55–68-day interval and ADG (− 0.70 ± 0.11). Intuitively, time spent lying and distance traveled are expected to have the strongest genetic correlations with ADG, as an animal that expends less energy is expected to grow faster. Obermier et al. [16] also found that pigs that spent more time lying and that were less active had higher growth rates and greater body weight at a given age. Sitting time was the behavior trait that was estimated to be least phenotypically and genetically correlated with all production traits, while distance was estimated to be the most correlated. As eating and drinking time only consider the amount of time spent at the feeder and waterer and not the quantity of feed or water consumed, it is expected that these traits are not strongly correlated with the production traits. The period with the strongest genetic correlations between behavior and production traits was the 55–68-day interval, and the period with the weakest correlations was the 27–40-day interval. Therefore, the last 2 to 3 weeks before off-test can best capture the relationship between behavior and production traits.

## Conclusions

Digital phenotyping for behavior traits recorded at an individual level provides an opportunity to further understand the association between behavior and production performance. Although behavior and production traits are not highly correlated, this study introduces the possibility of capturing behavior information and its potential association with production. Obtaining additional information on breeding candidates can increase accuracy of (G)EBV, leading to greater genetic improvement. The link between digital behavior data and economically relevant production traits is of interest, and digital phenotyping is a low-cost method to obtain this information on performance. High-throughput phenotyping is a new method for data collection; therefore, extensive quality control measures are needed before implementing the results into evaluations. The results of this study suggest that some pig behaviors, such as standing time, distance traveled, and laterally laying time, are phenotypically and genetically associated with ADG, BF, and LD. The behavior of animals 2 to 3 weeks before the off-test date had the strongest genetic correlations with the production traits. Digital phenotyping is promising for enhancing the efficiency, profitability, and rate of genetic gain in pig production.

## Data Availability

The dataset used in this study was obtained from a pre-existing dataset owned by Genus PIC.
